# Use of rasburicase to improve kidney function in children with hyperuricemia and acute kidney injury

**DOI:** 10.1007/s10157-023-02394-2

**Published:** 2023-09-26

**Authors:** María Herrero-Goñi, Amaia Zugazabeitia Irazábal, Leire Madariaga, Estibaliz Chávarri Gil, Leire Gondra, Mireia Aguirre Meñica

**Affiliations:** 1 Department of Pediatric Nephrology, Biocruces Bizkaia Health Research Institute, Cruces University Hospital, CIBERDEM, CIBERER, University of the Basque Country (UPV-EHU), Barakaldo, Bizkaia Spain; 2 Department of Pharmacy, Cruces University Hospital, Barakaldo, Bizkaia Spain; 3 Department of Pediatric Nephrology, Biocruces Bizkaia Health Research Institute, Cruces University Hospital, Barakaldo, Bizkaia Spain; 4 Department of Pediatrics, Biocruces Bizkaia Health Research Institute, Cruces University Hospital, Barakaldo, Bizkaia Spain

**Keywords:** Rasburicase, Acute kidney injury, Hyperuricemia, Pediatrics

## Abstract

**Background:**

Hyperuricemia contributes to decrease in kidney function and induces additional renal damage in children with acute kidney injury (AKI). Rasburicase oxidizes uric acid (UA), decreasing its serum quantities in less than 24 h.

**Methods:**

This is a retrospective study involving hospitalized patients under 18 years of age with underlying pathology diagnosed with AKI and severe hyperuricemia treated with rasburicase over a 4-year period.

**Results:**

We describe 15 patients from 4 days of life to 18 years (median: 4.4 years). Seventy-three percent had known underlying pathologies. All presented worsening of basal renal function or AKI data. All received the usual medical treatment for AKI without response. Twenty percent received an extrarenal depuration technique. All had hyperuricemia with a mean (± SD) of 13.1 (± 2.19) mg/dl. After rasburicase administration UA levels fell to a mean (± SD) of 0.76 (± 0.62) mg/dl (*p* < 0.001) in less than 24 h. In parallel, a decrease in the mean plasma creatinine was observed (2.92 mg/dl to 1.93 mg/dl (*p* = 0.057)) together with a significant improvement of the mean glomerular filtration rate (16.3 ml/min/1.73 m^2^ to 78.6 ml/min/1.73 m^2^) (*p* = 0.001)). No side effects were recorded. Kidney function normalized in all cases or returned to baseline levels.

**Conclusions:**

Although the use of rasburicase is not routinely approved in pediatric patients with severe hyperuricemia and AKI, it has been used successfully without complications, and helped prevent progressive kidney damage. This study could serve as a basis for suggesting the off-label use of rasburicase for the management of complex pediatric patients in whom UA plays an important role in the development of AKI.

**Supplementary Information:**

The online version contains supplementary material available at 10.1007/s10157-023-02394-2.

## Introduction

Hyperuricemia plays an important and well-documented role in acute and chronic kidney disease (CKD) [1, 2]. Increased production or decreased elimination of uric acid (UA) causes a supersaturation of tubular light that can result in the formation of UA crystals inducing renal obstruction resulting in oliguric acute kidney injury (AKI) [1]. UA nephropathy treatment often requires extrarenal depuration technique.

The pathogenic mechanisms by which UA can contribute to AKI are diverse, namely through renal vasoconstriction (via inhibition of nitric oxide synthase 1), antiangiogenic, proinflammatory and prooxidative processes, as well as alteration of renal autoregulation [1, 3].

This clinical picture should be suspected in situations such as hypovolemia or dehydration in patients with oligoanuric AKI with hyperuricemia and hyperuricosuria or at risk of tumor lysis syndrome (TLS) [3, 4].

UA nephropathy is well known in patients with cancer who develop TLS in the induction phase after chemotherapy or spontaneously if there is a large tumor burden or high proliferative rate [3, 5]. TLS is a consequence of a massive and accelerated destruction of malignant cells. The release of intracellular metabolites, including nucleic acids, proteins, phosphorus, and potassium, can overwhelm normal homeostatic mechanisms, potentially leading to hyperuricemia, hyperkalemia, hyperphosphatemia, hypocalcemia and uremia [6]. In some cases, TLS can lead to AKI or even death.

Rasburicase, unlike allopurinol, is a recombinant urate oxidase enzyme that oxidizes UA into a water soluble inactive metabolite, allantoin (Table 1) [6, 7]. Allopurinol has traditionally been used to reduce the cardiovascular risk factors associated with chronic hyperuricemia [8]. Allopurinol acts by inhibiting xanthine oxidase, prior to the formation of UA causing the accumulation of nephrotoxic precursors such as xanthine and hypoxanthine [9]. Consequently, it cannot lower the values of UA already present. Rasburicase, unlike allopurinol, works on existing UA (Table 1) [10, 11]. It is widely used in the prevention or treatment of TLS in children [10, 12].

**Table 1:** Main differences between rasburicase and allopurinol

	Allopurinol	Rasburicase
Effect on UA	Inhibits UA formation	Decreases UA levels
Onset of action	Days	Hours
Relative efficacy	Weak	Strong
Reported drug interactions	Azathioprine (among others)	None identified
Dose adjustments	Necessary in the setting of renal dysfunction	None
Black box warnings	None	Methemoglobinemia, anaphylaxis, hemolysis
Contraindications	None	G6PD deficiency
Available formulations	Endovenous and oral	Endovenous
Relative cost	Inexpensive	Expensive

*UA* Uric acid, *G6PD* Glucose-6-Phosphate dehydrogenase

Rasburicase has a very fast acting effect lowering the UA in a few hours [10]. No drug interactions have been reported and dose adjustment is not necessary in case of kidney dysfunction (unlike allopurinol). Its use is contraindicated in patients with glucose-6-phosphate dehydrogenase (G6PD) deficiency because it can produce methemoglobinemia and hemolytic anemia [7, 13]. If a G6PD deficiency is previously ruled out, few side effects have been reported. On one hand, some cases of anaphylaxis after repeated doses have been published [14, 15]. And on the other hand, the appearance of anti-rasburicase antibodies after repeated use has recently been described, leading to loss of therapeutic efficacy of the drug [16, 17].

The aim of our study was to analyze the effect of rasburicase in a cohort of pediatric patients with hyperuricemia and AKI to prevent the progression of kidney injury.

Second, to our knowledge, prior to this study, there is no documented experience with rasburicase in patients with underlying pathology. Its use in renal transplant recipients is especially novel and important due to the risk that hyperuricemia may pose to the kidney graft. Therefore, we analyzed the effect of rasburicase in patients with underlying pathology.

In addition, we have analyzed whether there are any side effects related to rasburicase administration in our cohort of patients.

## Materials and methods

### Study design

This is a descriptive study with retrospective data collection including patients under 18 years of age diagnosed with AKI and severe hyperuricemia treated with rasburicase. All were hospitalized in the Pediatric Nephrology Department at Cruces University Hospital (Vizcaya, Spain) over a 4-year period.

All patients had AKI and hyperuricemia with no response to medical treatment (24–48 h of serum therapy). Furthermore, all received treatment for their underlying pathology, without the rasburicase delaying or interfering with the administration of other medications.

### Clinical data

The following data were collected: age, sex, personal history, height/weight, the need for and duration of dialysis, extrarenal complications, death and cause of death. Blood results were collected previous to and 24 h after of rasburicase administration. We included: creatinine, urea, UA, sodium, chlorine, potassium, phosphorus, magnesium and calcium. Peak serum creatinine was also collected from all patients during hospital admission. We collected clinical data from patients’ medical records.

### Definitions: laboratory test and reference values

AKI has been defined as an increase in serum creatinine by ≥ 0.3 mg/dl within 48 h or by a urine volume of < 0.5 ml/kg/h for 6 h [18].

A patient was considered to have recovered baseline kidney function when creatinine decreased to the normal value for his or her height or to the value prior to admission plus 0.2 mg/dl. Glomerular filtration rate (GFR) was estimated using the Schwartz formula [18].

Severe hyperuricemia was defined as serum UA > 7.5 mg/dl [19].

Patients received an intravenous dose of 0.15 mg/kg rasburicase [12, 20] and UA levels were determined within 24 h of administration [12]. Sample processing after rasburicase administration was carried out in refrigerated heparinized tubes to minimize the ex vivo degradation of UA, and transported to the laboratory on ice for correct determination [21].

The outcome of the patients was divided into 3 groups: limitation of therapeutic effort, home discharge or exitus.

### Statistical analysis

Univariate statistical analyses were performed with SPSS 23 (IBM). A non-parametric paired t-test, Wilcoxon’s signed rank sum was used to determine significant differences. Continuous data were expressed as means ± standard deviations (SD) when they were normally distributed and as median and range when distribution was not normal. A *p* value of < 0.05 was considered to be statistically significant. Patient-specific information was de-identified.

### Consent

As the use of rasburicase for hyperuricemia in patients with AKI is off-label, all patients were verbally informed of its risks and benefits and written informed consent was obtained. There were no refusals.

Written informed consent was obtained from the patient (parent/legal guardian and children over 12 years of age) for the publication of this research.

Furthermore, this retrospective study involving human participants obtained approval from the Clinical Research Ethics Committee of Euskadi (ID: EOM2022024).

## Results

Rasburicase was administered to a total of 15 patients during the follow-up period. Clinical characteristics of these patients are shown in Table 2. The median age of the patients was 4.4 years (range 0 months to 18 years). Forty-seven percent were neonates with a mean ($\pm$ SD) gestational age of 36.28 (± 4.46) weeks. Eighty percent were male. Of the 15 patients, 66.7% were Caucasian, with 13.3% of Arab origin and 20% from Latin America.

**Table 2:** Clinical features in patient with hyperuricemia treated with rasburicase

Patient	Gender/ethnicity/age, months	Etiology	Underlying pathology	Uric acid (mg/dl) Day 0 (dose)/day 1 after rasburicase	Creatinine (mg/dl) Day 0 (dose)/day 1 in first 12 h/day 1 in first 24 h after rasburicase	Maximum creatinine (mg/dl) during hospital admission	Urine output (ml/kg/day) Day 0/day 1 in first 12 h/day 1 in first 24 h after rasburicase	Acute extrarenal depuration technique	Return to basal kidney function	Outcome
1	Male/Caucasian/15	Acute tubular necrosis	Severe pulmonary hypertension. Suspected metabolic disease	11.6/2.4	0.66/0.28/0.26	0.66	0.4/0.9/1.3	No	Yes	Limitation of therapeutic effort
2	Female/Latin American/6	Complex cardiopathy post-surgery	Complex cardiopathy	12.7*/1.2*	0.48*/0.21*/0.2*	0.63	0.4*/0.5*/0.7*	No	Yes	Home discharge
3	Female/Caucasian/194	Septic shock	Kidney transplant	9.6/0.5	4.16/3.19/2.7	4.5	0.3/0.9/1.4	No	Yes	Home discharge
4	Male/arabic/214	Acute tubular necrosis	Kidney transplant	15/0.1	12.3/4.13/3.5	15.6	0.3/1.4/2.1	No	Yes	Home discharge
5	Male/arabic/1	Complex cardiopathy post-surgery	Complex cardiopathy	11.5/0.4	0.35/0.22/0.2	0.41	0.7/1.3/1.6	Yes	Yes	Home discharge
6	Male/Latin American/1	Hypoxia	Gastroschisis	8.6/0.4	2.16/1.96/1.55	3.87	0.4/0.9/1.1	Yes	No	Home discharge
7	Male/Caucasian/0	Acute tubular necrosis	No	16.3/0.4	1.84/0.37/0.25	2.28	0.4/1/2.7	No	Yes	home discharge
8	Male/Caucasian/11	Acute tubular necrosis	Chronic kidney disease	13.8/1	2.92/1.6/1.5	4.4	0.2/1.8/2.5	No	Yes	Home discharge
9	Male/Caucasian/1	Complex cardiopathy post-surgery	Complex cardiopathy	16.3/0.4	1.14/0.92/0.6	1.14	0.7/1/1.9	Yes	Yes	Exitus
10	Male/Caucasian/76	Acute tubular necrosis	No	13.3/0.4	5.16/6.46/4.8	8.77	0.4/0.7/1.2	No	Yes	Home discharge
11	Female/Latin American/56	Hypoxia	Syndrome non-filiated	13.6/0.4	2.85/3.82/2.5	6.74	0.4/0.6/1	No	Yes	Home discharge
12	Male/Caucasian/0	Acute tubular necrosis	No	12.6/1	1.42/0.65/0.3	4.59	0.4/1.2/2.3	No	Yes	home discharge
13	Male/Caucasian/0	Acute tubular necrosis	No	14/1.8	1.4/0.45/0.28	3.89	0.3/1.5/2.1	No	Yes	Home discharge
14	Male/Caucasian/215	Acute tubular necrosis	Kidney transplant	12.6/0.5	5.53/2.82/2.02	7.12	0.5/0.9/1.5	No	Yes	Home discharge
15	Male/Caucasian/0	Septic shock	Complex cardiopathy, premature, bronchopulmonary dysplasia	15/0.5	1.44/1.95/1.5	3.11	0.8/1/1.8	No	No	Exitus

*These values express the mean after receiving repeated doses of rasburicase

All had severe hyperuricemia with a mean (± SD) UA of 13.1 (± 2.19) mg/dl (Table 3).

**Table 3:** Group changes after intravenous treatment for hyperuricemia with a dose of rasburicase on day 0

	Day 0, before rasburicase administration Mean (SD)	Day 1 after rasburicase Mean (SD)	*p* value
Creatinine (mg(dl)	2.92 (3.05)	1.93 (1.83)	0.057
Glomerular filtration rate (GFR) (ml/min/1.73 m^2^)	16.37 (18.26)	78.67 (60.11)	0.001
Urea (mg/dl)	120.80 (53.4)	98.60 (61.4)	0.028
Uric acid (mg/dl)	13.1 (2.19)	0.76 (0.62)	0.001
Sodium (mEq/L)	137.13 (8.24)	134.80 (6.34)	0.105
Potassium (mEq/L)	4.31 (1.1)	6.43 (9.6)	0.308
Chlorine (mEq/L)	100.87 (11.26)	101.00 (9.07)	0.729
Phosphorus (mg/dl)	7.10 (1.66)	5.69 (1.61)	0.02
Calcium (mg/dl)	9.3 (0.88)	9.02 (0.89)	0.177
Magnesium (mg/dl)	2.67 (1.38)	2.03 (0.77)	0.009

Data are expressed as means (SD)

*SD* standard deviation, *N*S Non-sense

All patients presented worsening of basal renal function or AKI data with a mean (± SD) GFR of 16.3 (± 18.2) ml/min/1.73 m^2^. Seventy-three percent (73.3%) had known underlying pathologies, of which 20% were kidney transplants, 20% postoperative complex congenital heart disease (CHD), 6.7% CKD and 26.7% other pathologies. However, these patients with prior baseline pathologies showed no relevant difference in renal function compared with the rest (mean serum creatinine 3.09 mg/dl *vs*. 2.45 mg/dl, *p* = 0.73).

The cause of AKI for these patients was: 53.3% acute tubular necrosis (ATN) in the context of severe dehydration, 13.3% due to hypoxia, 13.3% in the context of septic shock and 20% of multifactorial origin in the postoperative period of a complex CHD.

**Fig. 1:**
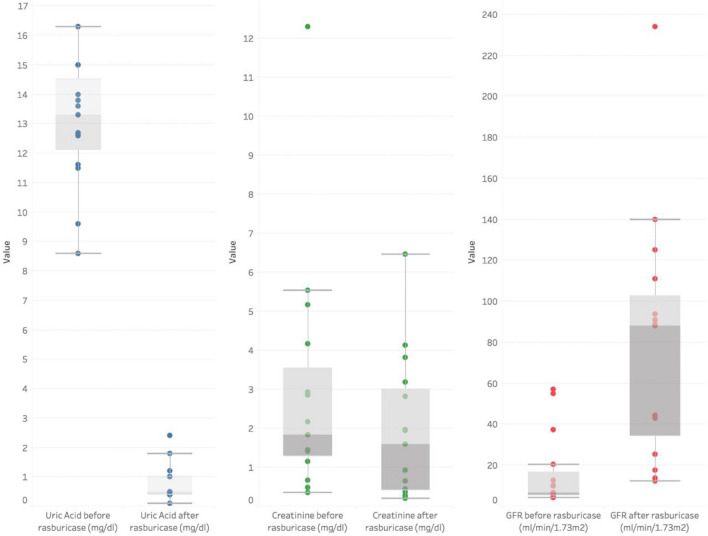
Changes in uric acid, creatinine and glomerular filtration rate. Changes in uric acid, creatinine and glomerular filtration rate in the group after administration of a single dose of rasburicase on day 0

**Fig. 2:**
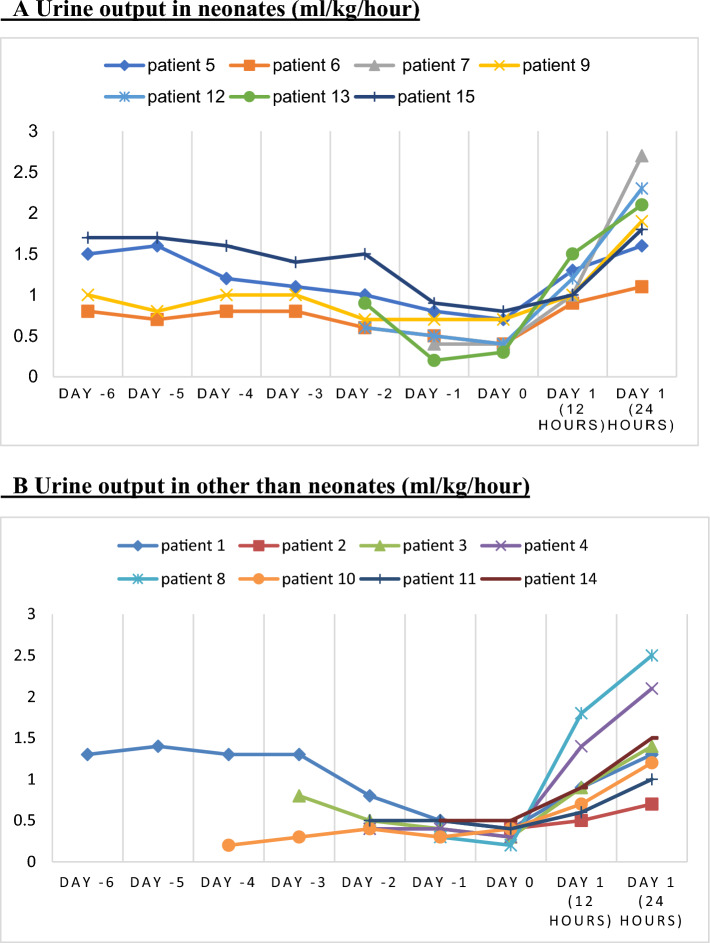
**A** Urine output in neonates (ml/kg/hour). **B** Urine output in other than neonates (ml/kg/hour). Graphic shows urine output (ml/kg/h) in the days prior to rasburicase administration (from 6 days before to the day of rasburicase administration), the day of rasburicase administration (day 0) and the first two determinations (in the first 24 h) after administration, in neonates (Fig. 2A) and other than neonates (Fig. 2B). The values of patient number 2 express the mean after receiving repeated doses of rasburicase

They all received medical treatment for hyperuricemia and AKI: serum therapy and/or diuretics depending on the clinical situation of the patient. In 20%, an extrarenal depuration technique was also used for acute liquid handling or correction of electrolyte disturbances. No patient required chronic dialysis (Table 2). In addition, once medical treatment had been administered, they were all treated with rasburicase. After treatment with rasburicase, mean (± SD) UA levels decreased to 0.76 (± 0.62) mg/dl in the first 24 h (*p* 0.001). As shown in Online Resource 1, an initial decrease in creatinine was obtained in patients with the usual medical treatment (fluid and electrolyte correction). After administration of rasburicase on day 0, an improvement in serum creatinine was obtained in all patients in the first 24 h. Mean (± SD) serum creatinine levels decreased from 2.92 mg/dl (± 3.05) before rasburicase administration to 1.93 mg/dl (± 1.83) (*p* 0.057). In this sense, there was also a significant improvement of the mean GFR (16.3 ml/min/1.73 m^2^ to 78.6 ml/min/1.73 m^2^ (*p* 0.001)) (Table 3, Figure 1 and Online Resource 1). In neonates, we also observed a significant increase in mean GFR (15.3 ml/min/1.73 m^2^ to 86 ml/min/1.73 m^2^ (*p* 0.018)). Mean blood urea levels were 120.80 mg/dl pre-administration and 98.60 mg/dl post-administration (*p* 0.028) (Table 3). Parallel to the improvement in kidney function, all patients showed oliguria prior to rasburicase administration, with an increase of the diuresis rate in the first hours after rasburicase administration (Table 2, Fig. 2).

There were significant differences in magnesium and phosphorous levels measured before and after administration (*p* < 0.01 and *p* 0.02, respectively). No patient received medical treatment for hyperphosphatemia. Differences in sodium, chlorine, potassium and calcium levels before and after rasburicase administration were not significant (Table 3).

Patient 2 received rasburicase 5 times (Table 4) in the context of refractory cardiogenic shock with severe heart and respiratory failure in the postoperative period of a complex heart disease that required extracorporeal cardiac support. This patient remained in hospital for 16 months. After administration of rasburicase, the mean UA levels decreased from 12.7 to 1.2 mg/dl (*p* 0.001) and the mean creatinine value normalized (0.48 mg/dl pre-administration *vs*. 0.21 mg/dl post-administration). The patient had no reported side effects related to repeated administration of rasburicase.

**Table 4:** Repeated doses of rasburicase in patient number 2

Rasburicase administration	Uric acid before/after rasburicase (mg/dl)	Creatinine before/after rasburicase (mg/dl)
1	10.7/ < 0.5	0.53/0.21
2	12/1.9	0.44/0.23
3	13.1/3.1	0.45/0.12
4	13.8/0.5	0.57/0.3
5	14.2/ < 0.5	0.43/0.2

Finally, rasburicase was well tolerated by all the patients and no treatment-related adverse events were observed. Two patients died during follow-up due to their underlying disease: therapeutic effort was limited in one patient with a metabolic disease (patient 9) and another died in the postoperative phase of a complex CHD (patient 15).

Before discharge, 86% recovered their basal kidney function (Table 2). One patient (patient 6) was discharged with CKD. The other patient who did not recover his basal kidney function was patient 15 who died in the postoperative period from his complex heart disease.

## Discussion

In this study, we analyzed the effects of rasburicase in pediatric patients (from neonates to 18 years old) diagnosed with AKI and severe hyperuricemia. As the most relevant and novel aspect, all patients administered rasburicase had an underlying disease. Administration of rasburicase was effective in 100% of patients, achieving a significant reduction of UA levels in the first 24 h and subsequent improvement of renal function. Complete renal functional recovery was obtained in most patients (Table 3).

Hyperuricemia is not only a marker involved in the development of renal damage but also worsens a previous underlying nephropathy [1, 2]. Furthermore, UA is implicated in the pathogenesis of AKI through renal vasoconstriction, inflammatory mechanisms, microvascular injury, disturbances in renal autoregulation and renal obstruction leading to ATN [1].

Our study is, to our knowledge, the first to report the use of rasburicase in hyperuricemic children with kidney failure and an underlying disease such as a CHD, kidney transplant or metabolic disease.

The treatment of acute hyperuricemia is classically based on aggressive intravenous hydration to improve renal perfusion and glomerular filtration, inducing a high urine output to minimize the likelihood of UA or calcium phosphate precipitation in the tubules [4, 5]. However, intravenous hydration can be dangerous in patients with underlying disease such as renal disease or cardiac dysfunction as in our series. Although urine alkalinizing agents have also been used in severe hyperuricemia, there are no consistent studies demonstrating their efficacy [5, 22]. In addition, alkalinization to a urine pH of 6.5 to 7 or even higher may be dangerous because it promotes calcium phosphate deposition in the kidney especially in patients who develop marked hyperphosphatemia [5, 22]. In our series of patients, acute hyperuricemia was initially treated with hydration and/or diuretics according to the underlying disease. However, this therapy was not enough to obtain the desired decrease in UA to avoid a decrease in kidney function.

Besides rasburicase, other drugs have been used to treat acute hyperuricemia, namely allopurinol and febuxostat [23]. The first has been used to slow the progression of renal disease through the lowering of UA levels. However, several studies have not been shown to be effective in eliminating the UA already present, which causes accumulation of nephrotoxic precursors [1, 2, 6, 7]. Goldman et al. [24] compared in a clinical trial the effectiveness of rasburicase and allopurinol in hyperuricemic patients showing an improvement in GFR in patients who had received rasburicase and a worsening after the use of allopurinol. The latter has been proposed for the treatment of acute hyperuricemia when allopurinol is not tolerated, and rasburicase is not available or is contraindicated. Furthermore, it is only approved in adults over 18 years of age. Therefore, we did not consider its administration in these patients for the treatment of hyperuricemia [23]. We chose rasburicase, which has been widely used safely for over 25 years for TLS [5–7, 12].

In our series, the administration of rasburicase was effective in 100% of patients, achieving a significant reduction in UA levels in the first 24 h. In all of them, after the decrease of UA, the creatinine levels decreased with a progressive increase of the GFR reaching a complete renal recovery in most patients (Table 3 and Online Resource 1). As previously mentioned, hyperuricemia plays an important role in the progression of renal damage through proinflammatory mechanisms, microvascular injury and tubular obstruction [1, 2]. Therefore, we consider that the improvement in patients’ renal function may be related to the decrease in UA and the probable decrease in the risk of tubular obstruction and thus ATN.

As shown in Online Resource 1, a decrease in creatinine in the first 24 h after rasburicase administration (day 0) was observed in all patients in parallel to the decrease in UA levels. As mentioned above, rasburicase was administered after optimizing the usual medical treatment. In this sense, the initial decrease in creatinine (the days prior to rasburicase administration) can be explained because the patients received conservative treatment (fluid and electrolyte correction) for renal damage during this period. All patients, despite having received medical treatment, had oliguria before rasburicase administration with a significant increase in the rate of diuresis in the hours following rasburicase administration (Fig. 2, Table 2). This change in diuresis rate can be considered a good marker of AKI improvement which supports that rasburicase contributes to the improvement of kidney function in these patients. Patient 11 had a slower improvement of renal function probably because she had a severe post-cardiac arrest syndrome.

There is no documented experience of rasburicase in patients with underlying pathology. Almost all articles focus on neonates and infants [25–28] and its use in hemolytic uremic syndrome has recently been published [26, 29, 30]. In our series, positive results were seen in complex high-risk patients with a wide range of pathologies (previous kidney transplant or CKD among others) or in cases of difficult-to-manage multifactorial AKI (as in postoperative CHD). We also consider that its use in renal transplant patients is especially novel and important due to the risk that hyperuricemia may represent in the renal graft, even leading to the loss of kidney function.

Given the complexity of patients in a life-threatening clinical situation, it is difficult to predict what would had happened if rasburicase had not been administered. However, its use was successful and helped to prevent the progression of kidney injury. Therefore, the use in such complex patients, together with the absence of side effects, reinforces the suitability of rasburicase use.

Regarding side effects described with the use of rasburicase, there is ample evidence that it induces methemoglobinemia in patients with G6PD deficiency [13]. In our series no patient developed hemolysis because the G6DP deficiency was previously ruled out in all of them.

In addition, two infrequent but important complications have been described with the repeated use of rasburicase: anaphylactic reactions [14] and the appearance of anti-rasburicase antibodies that cause a loss of efficacy of the drug [16, 17]. According to previous studies, rasburicase can induce anaphylaxis in up to 6% of patients [14]. The risk immunogenicity is increased with higher drug doses, with a longer duration of treatment, repeated doses and in intravenous administration [15]. Up to 64% of patients can develop antibodies within 1–6 weeks [14]. However, no studies have assessed the correlation between antibody formation and anaphylactic reactions. In our series, patient 2 received rasburicase on 5 occasions during the 16 months she was hospitalized (Table 4). After administration of rasburicase, UA decreased every time and she presented no allergic symptoms. Although pre-medication was not needed, we considered the used of antihistamines and corticosteroids, and if necessary, epinephrine pre-rasburicase.

In conclusion, in our series, there were no complications attributable to repeated doses; neither allergic events nor lack of response to its administration that would have suggested the appearance of anti-rasburicase antibodies. The need for repeated doses highlights its deleterious effect if the situation giving rise to hyperuricemia persists over time. Moreover, it opens the door to its repeated use without noticeable side effects if the use of pre-medication is considered.

Electrolytic imbalances following treatment of acute hyperuricemia are a matter of concern in these patients with severe pathologies. However, we found no differences in sodium, potassium, chlorine and calcium concentration after rasburicase administration in our patients, probably because they were admitted to the intensive care unit where ions are frequently determined and continuously supplemented or replenished in advance. In addition, magnesium and phosphorus levels significantly decreased after rasburicase administration (Table 3) in relation to the improvement in renal function experienced after the treatment [3]. Phosphorus monitoring is important because hyperphosphatemia in relation to renal failure can lead to renal phosphorus precipitation and induce nephropathy [3].

This study has certain limitations. First, the data were collected retrospectively. However, the fact that all the cases seen in our department are recorded electronically, including medical records and notes on the evolution and follow-up of the patients, makes the collection process consistent. Secondly, the small sample size limits the drawing of conclusions and therefore the generalization of rasburicase in pediatric patients with AKI and hyperuricemia. Furthermore, since this was not a multicenter study, it is difficult to extrapolate our findings to other areas. Studies with larger sample sizes that can support the beneficial effect of rasburicase are needed. Although this was not the main objective of this study, larger studies would facilitate the drawing of conclusions by patient subgroups according to their underlying pathology.

In conclusion, although rasburicase is not routinely approved in pediatric patients with AKI, in our series, its use has led to an improvement of kidney function. Furthermore, we have observed no side effects or complications associated with its administration even in patients with underlying diseases. For all these reasons, and given that there is currently no effective therapeutic alternative in patients with hyperuricemia and AKI, we suggest its off-label use. However, studies with larger sample sizes are needed to support these findings.

### Supplementary Information

Below is the link to the electronic supplementary material.

Supplementary file1 (DOCX 50 KB)

## Data Availability

The data analyzed during the current study are available from the corresponding author on reasonable request.

## Abbreviations

AKI: Acute kidney injury
UA: Uric acid
TLS: Tumor lysis syndrome
GFR: Glomerular filtration rate
CKD: Chronic kidney disease
G6PD: Glucose-6-phosphate dehydrogenase
SD: Standard deviations
CHD: Congenital heart disease
ATN: Acute tubular necrosis

## Declarations

### Data transparency

All the authors make sure that all data and materials support the published claims and comply with field standards.

## References

[1] Ejaz AA, Johnson RJ, Shimada M, Mohandas R, Alquadan KF (2019). The role of uric acid in acute kidney injury. Nephron.

[2] Fathallah-Shaykh SA, Cramer MT (2014). Uric acid and the kidney. Pediatr Nephrol.

[3] Howard SC, Jones DP, Pui CH (2011). The tumor lysis syndrome. N Engl J Med.

[4] Cairo MS, Coiffier B, Reiter A, Younes A (2010). TLS Expert Panel. Recommendations for the evaluation of risk and prophylaxis of tumour lysis syndrome (TLS) in adults and children with malignant diseases: an expert TLS panel consensus. Br J Haematol.

[5] Coiffier B, Altman A, Pui CH, Younes A, Cairo MS (2008). Guidelines for the management of pediatric and adult tumor lysis syndrome: an evidence-based review. J Clin Oncol.

[6] Criscuolo M, Fianchi L, Dragonetti G, Pagano L (2016). Tumor lysis syndrome: review of pathogenesis, risk factors and management of a medical emergency. Expert Rev Hematol.

[7] Yim BT, Sims-McCallum RP, Chong PH (2003). Rasburicase for the treatment and prevention of hyperuricemia. Ann Pharmacother.

[8] Siu YP, Leung KT, Tong MK, Kwan TH (2006). Use of allopurinol in slowing the progression of renal disease through its ability to lower serum uric acid level. Am J Kidney Dis.

[9] LaRosa C, McMullen L, Bakdash S, Ellis D, Krishnamurti L (2007). Acute renal failure from xanthine nephropathy during management of acute leukemia. Pediatr Nephrol.

[10] Cairo MS, Thompson S, Tangirala K, Eaddy MT (2016). A clinical and economic comparison of rasburicase and allopurinol in the treatment of patients with clinical or laboratory tumor lysis syndrome. Clin Lymphoma Myeloma Leuk.

[11] Martens KL, Khalighi PR, Li S, White AA, Silgard E (2020). Comparative effectiveness of rasburicase versus allopurinol for cancer patients with renal dysfunction and hyperuricemia. Leuk Res.

[12] Kikuchi A, Kigasawa H, Tsurusawa M, Kawa K, Kikuta A (2009). A study of rasburicase for the management of hyperuricemia in pediatric patients with newly diagnosed hematologic malignancies at high risk for tumor lysis syndrome. Int J Hematol.

[13] Raru Y, Abouzid M, Parsons J, Zeid F (2018). Rasburicase induced severe hemolysis and methemoglobinemia in a Caucasian patient complicated by acute renal failure and ARDS. Respir Med Case Rep.

[14] Allen KC, Champlain AH, Cotliar JA, Belknap SM, West DP (2015). Risk of anaphylaxis with repeated courses of rasburicase: a Research on Adverse Drug Events and Reports (RADAR) project. Drug Saf.

[15] Kessler M, Goldsmith D, Schellekens H (2006). Immunogenicity of biopharmaceuticals. Nephrol Dial Transplant.

[16] Xu H, Feldman GM, Max EE (2020). High-dose IV administration of rasburicase suppresses anti-rasburicase antibodies, depletes rasburicase-specific lymphocytes, and upregulates Treg cells. AAPS J.

[17] Moia R, Boggio E, Gigliotti L, Crisà E, De Paoli L (2020). Anti-rasburicase antibodies induce clinical refractoriness by inhibiting the enzyme catalytic activity. Hematol Oncol.

[18] Sutherland SM, Byrnes JJ, Kothari M, Longhurst CA, Dutta S (2015). AKI in hospitalized children: comparing the pRIFLE, AKIN, and KDIGO definitions. Clin J Am Soc Nephrol.

[19] Johnson RJ, Bakris GL, Borghi C, Chonchol MB, Feldman D (2018). Hyperuricemia, acute and chronic kidney disease, hypertension, and cardiovascular disease: report of a scientific workshop organized by the national kidney foundation. Am J Kidney Dis.

[20] Taketomo CK, Hodding JH, Kraus DM (2018). Rasburicase. Pediatric and Neonatal Dosage Handbook.

[21] European Medicines Agency (EMA). 2009 [updated 2020].Fasturtec. Available at:https://www.ema.europa.eu/en/documents/overview/fasturtec-epar-summary-public_en.pdf.

[22] Cairo MS, Bishop M (2004). Tumour lysis syndrome: new therapeutic strategies and classification. Br J Haematol.

[23] Matuszkiewicz-Rowinska J, Malyszko J (2020). Prevention and treatment of tumor lysis syndrome in the era of onco-nephrology progress. Kidney Blood Press Res.

[24] Goldman SC, Holcenberg JS, Finklestein JZ, Hutchinson R, Kreissman S (2001). A randomized comparison between rasburicase and allopurinol in children with lymphoma or leukemia at high risk for tumor lysis. Blood.

[25] Hobbs DJ, Steinke JM, Chung JY, Barletta GM, Bunchman TE (2010). Rasburicase improves hyperuricemia in infants with acute kidney injury. Pediatr Nephrol.

[26] Cho MH, Ahn YH, Lim SH, Kim JH, Ha IS (2020). Rasburicase improves the outcome of acute kidney injury from typical hemolytic uremic syndrome. Pediatr Nephrol.

[27] Ghirardello S, Ardissino G, Mastrangelo A, Mosca F (2010). Rasburicase in the treatment of hyperuricemia of newborns. Pediatr Nephrol.

[28] Canpolat FE, Cekmez F (2011). Rasburicase for hyperuricemia in an extremely low birth weight infant. Indian Pediatr.

[29] Acosta AA, Hogg RJ (2012). Rasburicase for hyperuricemia in hemolytic uremic syndrome. Pediatr Nephrol.

[30] Balestracci A, Meni Battaglia L, Martin SM, Toledo I (2020). Rasburicase in hemolytic uremic syndrome related to Shiga toxin-producing Escherichia coli: a report of nine cases. Pediatr Nephrol.

