# Supplementary material for: Use of rasburicase to improve kidney function in children with hyperuricemia and acute kidney injury
Source: Clin Exp Nephrol. 2023 Sep 26;28(1):13–22. doi: 10.1007/s10157-023-02394-2 (PMC10766662; doi:10.1007/s10157-023-02394-2)
Supplement: Supplementary file 1 — Supplementary file1 (DOCX 50 KB) [file 10157_2023_2394_MOESM1_ESM.docx]

**TITLE PAGE**

**TITLE**

Use of Rasburicase to Improve Kidney Function in Children with Hyperuricemia and Acute Kidney Injury

**JOURNAL NAME**

Clinical and Experimental Nephrology

**AUTHOR INFORMATION:**

- María Herrero-Goñi. Department of Pediatric Nephrology. Biocruces Bizkaia Health Research Institute, Cruces University Hospital. Barakaldo, Bizkaia, Spain.
  - ORCID: 0000-0001-6501-0830
  - Author for correspondence: [maria.herrerogoni@osakidetza.eus](mailto:maria.herrerogoni@osakidetza.eus)
- Amaia Zugazabeitia Irazábal. Department of Pediatrics. Biocruces Bizkaia Health Research Institute, Cruces University Hospital. Barakaldo, Bizkaia, Spain.
  - ORCID: 0000-0002-5030-3179
- Leire Madariaga. Department of Pediatric Nephrology, Biocruces Bizkaia Health Research Institute, Cruces University Hospital, CIBERDEM, CIBERER, University of the Basque Country (UPV-EHU). Barakaldo, Bizkaia, Spain.
  - ORCID: 0000-0002-4032-9842
- Estibaliz Chávarri Gil. Department of Pharmacy, Cruces University Hospital. Barakaldo, Bizkaia, Spain.
- Leire Gondra. Department of Pediatric Nephrology, Biocruces Bizkaia Health Research Institute, Cruces University Hospital, CIBERDEM, CIBERER, University of the Basque Country (UPV-EHU). Barakaldo, Bizkaia, Spain.
  - ORCID: 0000-0003-3848-0196
- Mireia Aguirre Meñica. Department of Pediatric Nephrology, Biocruces Bizkaia Health Research Institute, Cruces University Hospital. Barakaldo, Bizkaia, Spain.

**Online Resource 1**

**1A creatinine (mg/dl) in neonates**

**1A uric acid (mg/dl) in neonates**

**1B creatinine (mg/dl) in other than neonates**

**1B uric acid (mg/dl) in other than neonates**

Graphic shows creatinine (mg/dl) and uric acid (mg/dl) in the days prior to rasburicase administration (from 6 days before to the day of rasburicase administration), the day of rasburicase administration (day 0) and the first two determinations (in the first 24 hours) after administration, in neonates (1A) and other than neonates (1B). There are no creatinine data in the days prior to rasburicase administration in those patients in whom blood tests were not performed. Patient 2 is excluded because received repeated doses of rasburicase.
